# Semantic modelling of animal welfare explained – Part 2: The basis of welfare weighting and usage of scientific information

**DOI:** 10.5194/aab-69-383-2026

**Published:** 2026-07-16

**Authors:** Margret L. Vonholdt-Wenker, Janine Benthin, Karen Kauselmann, Marc B. M. Bracke, E. Tobias Krause

**Affiliations:** 1 Institute of Animal Welfare and Animal Husbandry, Friedrich-Loeffler-Institute, Celle, 29223, Germany; 2 Wageningen Livestock Research, Wageningen University and Research, Wageningen, 6700 AH, the Netherlands

## Abstract

A methodology called semantic modelling can be used to synthesise scientific information to assess farm animal welfare. Different housing systems consist of attributes that can each have an effect on the welfare of the animal. Examples include type of floor, water provision system, and lighting system. Each of those attributes has so-called attribute levels. So, for instance, a water provision system can be drinking nipples, bowls, or troughs. Statements from the scientific literature that report the welfare effects of certain attribute levels serve as the basis for the welfare assessment. A semantic model takes attribute levels describing a housing system as input and generates a weighted overall welfare score as output. Although the procedure used in semantic modelling to decompose scientific statements into welfare-relevant attributes has been applied to multiple contexts, the procedure of information extraction has not been described in sufficient detail to allow new modellers to easily understand the procedure. Hence, the reproducibility of semantic modelling may be at stake despite its potential for integrated animal welfare research. Therefore, the objective of this paper was to provide a set of formalised rules showing how scientific information can be decomposed and weighted using semantic-modelling principles. We describe how to read the scientific literature to select scientific statements and how to decompose them to extract the relevant information for building a semantic model. Statement decomposition entails that the original formulation of the scientific statement is transformed into an “if–then” rule using this format: “if attribute A's level L1 is compared to its level L2 then there is a ([large] [significant]) effect on welfare measure M, with M belonging to a specified category of measures used for weighting (a so-called weighting category, WCat)”. The attribute levels are then weighted based on the incidence, duration, and intensity of the measured welfare effect, expressed by a so-called weighting-category level score (WCatLevSc). The guidelines presented in this paper could be an important step towards the transparent use of available scientific information for sustainable development supporting both human and animal welfare.

## 1 Introduction

Citizens' demands for improved farm animal welfare are becoming increasingly urgent worldwide (European Commission, 2023; Buller et al., 2018). It is an important and relevant topic that drives research. For several decades, applied animal scientists have generated a growing body of scientific knowledge about animal behaviour, stress, health, and production parameters related to farm animal husbandry systems. For science-based policy-making, the available scientific information should be compiled. Performing a meta-analysis or systematic review is one established way to synthesise multivariate data. However, those efforts have thus far been performed for and are probably suited to only a limited number of animal welfare topics, e.g. different floor types in beef cattle (Keane et al., 2018), enrichment materials in laying hens (Van Staaveren et al., 2021), or the combination of both for fattening pigs (Averós et al., 2010). Semantic modelling is an alternative approach that is suitable to provide a more generalised synthesis in the new area of integrated animal welfare research. It concerns a procedure to assess overall welfare using a wider range of the available knowledge in a transparent and “best-possible” way based on a systematic decomposition of the literature (Bracke, 2001). Scientific statements typically describe how different treatments affect the welfare of animals. This information can be used to rank (aspects of) housing systems and to identify knowledge gaps. So far, semantic modelling has been applied for overall welfare assessment of housing systems for several species of farm animals including pregnant sows (Bracke et al., 2002a, b), laying hens (Mol et al., 2006; Shimmura et al., 2011), dairy cows (Ursinus et al., 2009), and Atlantic salmon (Tschirren et al., 2021; Stien et al., 2013; Pettersen et al., 2014; Folkedal et al., 2016). In addition, semantic models have been used to assess specific welfare issues, such as the risk of tail biting in pigs (Bracke et al., 2004a, b) and the welfare value of enrichment materials for growing pigs (Bracke et al., 2006; Bracke, 2008).

Semantic modelling of animal welfare differs from semantic modelling in computer science (Rishe, 1992). The former derives its name from the role semantics, i.e. the meaning of words in scientific statements, plays in deriving overall welfare scores. The attention to the meaning of words is also illustrated by the fact that animal welfare is defined as the quality of life as perceived by the animals themselves (Bracke et al., 1999a). This is a feelings-based conception of animal welfare (Fraser et al., 1997). It is implied by the meaning of the word welfare, which refers to “faring well”, i.e. animal happiness (Webb et al., 2019). While feelings define welfare, the biological-functioning conception – and, to a limited, extent also the natural-living conception – identified by Fraser et al. (1997) is used in the semantic-modelling procedures to operationalise welfare assessment. These procedures have thus been developed based on an understanding of the meaning (semantics) of the available science, where peer-reviewed papers provide the norm. Housing and management systems have been studied using animal-based welfare measures. The scientific literature thus describes how environment-based measures affect the animal's welfare (Bracke et al., 1999b, 2001). Based on the systematic decomposition of scientific statements, a semantic model can be generated with a set of welfare-relevant attributes. These are welfare-relevant properties of a housing system. Each attribute has two or more levels and a weighting factor (WF) that allow for the calculation of a weighted overall welfare score (OWSc) on a scale from 0 to 1 as output (for a glossary see Table 1).

**Table 1:** Glossary of terms used in semantic modelling of animal welfare.

Key term	Definition
Animal welfare	The quality of life as perceived by the animals themselves (Bracke et al., 1999a). The welfare status is assessed by the degree of satisfaction and frustration of the animal's welfare needs (this term is described below).
Assessment domain	The range of housing systems for the species and production direction of interest that can be assessed by the model. The assessment domain specifies, for example, that the model is made for pregnant sows or for laying hens and only for systems that meet legal requirements or only for organic systems.
Attribute	Welfare-relevant variable of a housing system that fulfils or otherwise may help to fulfil a need, e.g. enrichment material (to fulfil the need for exploration) or space per pen (for movement).
Attribute level	Welfare-relevant property of a housing system, i.e. technical specification of a welfare-relevant attribute in a housing system (e.g. three levels of the attribute of enrichment material could be straw, metal chain, or no enrichment material available; an attribute level of space per pen could be 2–3 $m^{2}$ per pig). Together, the attribute levels define the welfare-relevant properties of the housing and management system in the assessment domain. Attribute levels are derived from the scientific statements obtained from the literature.
Attribute level score (ALevSc)	A score that reflects the ranking of the levels of an attribute, where the worst level receives a score of 0 and the best level receives a score of 1, with intermediate levels receiving intermediate ALevScs in direct proportion to their ranks. Like the attribute levels themselves, their ranks also result from (the decomposition of) the scientific statements. For example the following statement “… the available scientific evidence indicates that metal objects are not suitable enrichment materials for pigs, … and that straw and compound materials are best” (cited from Bracke et al. (2006), p. 166) generates the attribute levels of “straw” and “compound material”, which have a higher rank and thus a higher ALevSc than the level of metal object.
Decomposition	Translation of a scientific statement into welfare components as explained in more detail in this paper. This follows the procedure of breaking down a scientific statement into an if–then rule to extract the welfare-relevant components for semantic modelling. The components are attributes (e.g. enrichment material), attribute levels (e.g. straw, metal chain), weighting categories (WCats, e.g. preferences, abnormal behaviour), WCat level scores (see below), and welfare needs (e.g. exploration).
Housing system	Also called the “housing and management system”, i.e. the living conditions (environment) of animals defined through physical objects (floor, walls, feeders, etc.) and management practices (e.g. feeding, mixing, health care). The “housing system” thus, in principle, also includes the animals (conspecifics), as well as the caretaker(s) (farmer/owner) and his/her management decisions. The scope of the “housing system” may, however, be restricted by the assessment domain. This is the case when the model is, for example, designed to apply only to systems with good management practices or to systems with commercial food provision (thus excluding states of starvation to a degree that may occur in the wild).
Overall welfare score (OWSc)	Weighted average of all attribute level scores of a housing system, i.e. sum of all ALevScs multiplied by the weighting factor (WF) of each attribute and divided by the sum of all of the WFs (of all of the attributes) in the model (for more details, see Part 1 (Benthin et al., 2026)).
Scientific statement	Sentence or section of the scientific literature that typically describes a relationship between environment-based welfare input and animal-based welfare output. For example “Sows are social animals that live in groups of between two and four adult animals (Graves, 1984)”. In this statement the welfare input is the (semi-)natural environment. The welfare output is the observation that sows will organise themselves in social groups when given the opportunity. The weighting category here is natural behaviour. The welfare need is social contact. The welfare-relevant attribute is group size, and the main attribute level is “two to four adult animals” (which is implicitly compared to solitary living and living in (very) large groups, which are group sizes apparently not in accordance with commonly observed natural behaviour of sows).
Semantic modelling	A systematic, formalised procedure that aims to provide a quantified assessment of overall animal welfare based on all or as much as possible of the relevant scientific literature. A proper understanding of the meaning of words and scientific statements (and the common conceptual framework for welfare assessment as described in Anonymous, 2001) plays a crucial role in the translation of scientific information into welfare scores, hence the term “semantic”.

**Table 1:** Continued.

Key term	Definition
Type of welfare measure	Specification of the welfare parameter, e.g. plasma cortisol level, time spent on dust bathing, prevalence of mastitis. The term “type” is used to distinguish different welfare measures that are classified together in the same so-called weighting category (see next item in the glossary).
Weighting category (WCat)	Class of welfare measures. In total 12 WCats have been identified, including 3 WCats to assess positive welfare (i.e. natural behaviour, preference, and demand) and 9 for negative welfare (i.e. pain, illness, productivity and fitness, survival, aggression, stress physiology (hypothalamic-pituitary–adrenal) axis and sympathetic adreno-medullar axis activation (HPA and SAM, respectively), abnormal behaviour, and frustration and avoidance; see also Table 2).
Weighting-category level score (WCatLevSc)	A score that reflects the impact on welfare of one attribute level compared to another in virtue of (the meaning of) the scientific statement. For example, scientific statements reporting a strong preference for straw, social contact, or food generate higher WCatLevScs than statements reporting mere preferences or even indifference. The WCatLevScs of different WCats are added to generate an attribute's WF.
Weighting factor (WF)	The relative welfare importance of an attribute as a whole compared to other attributes that, together, make up the welfare model. The WF is derived by comparing the best and worst levels of an attribute and their maximum positive and negative WCatLevScs per WCat and the number of types (for more details see Benthin et al., 2026).
(Welfare) need	Needs are “requirements, which are a consequence of the biology of the animal to obtain a particular resource or respond to a particular environmental or bodily stimulus” (Broom and Johnson, 1993). In semantic modelling a welfare need is a component of welfare (quality of life) in virtue of expressing the state of a cognitive–emotional control mechanism that has evolved over the course of evolution to deal with a variable (and challenging) environment (Wiepkema, 1987). Examples of welfare needs are the need for food, water, rest, exploration, and social contact. Each welfare need refers to a behavioural system (which is typically goal- or resource-directed, e.g. food searching and ingestion); a physiological system (e.g. digestion), often with typical patho-physiology (e.g. emaciation in the case of starvation and obesity in the case of over-consumption); and, most importantly for welfare assessment, a range of associated (more or less) positive and negative affective states or feelings expressing different need states ranging from need frustration (e.g. being very hungry) to need satisfaction (feeling satiated).

Despite the various scientific publications on sematic modelling of animal welfare, further formalisation of scientific information processing is needed to generate better welfare scores. We examined the existing models during the development of the ANyWEL model framework in the InKalkTier project, aiming for integrated sustainability assessment (i.e. complementing welfare assessment with economic and environmental (emission) data (Benthin et al., 2023; KTBL, 2024). The ANyWEL model framework was designed to assess the welfare of any type of (farm) animal. It is described in detail in our companion paper (Part 1; see Benthin et al., 2026). During the development of that framework, we discovered that the basic procedure of statement decomposition as used in semantic modelling was not easy to understand for new modellers. Hence, these procedures needed further explanation and formalisation. Preliminary reliability trials showed a robust intra-modeller agreement with a reliability of about 80 %, while the inter-modeller reliability was substantially lower (about 40 %–54 %; see Supplement File S1). Hence, the reproducibility of semantic modelling (and existing welfare models) may be at stake despite its relevance and robust outcomes in validation studies (Bracke et al., 2002b, 2004b, 2007). A lack of reproducibility can slow down scientific progress (Open Science Collaboration, 2015), erode public trust, and have negative implications for stakeholders and policy-makers (Munafò et al., 2017). This generated a need for more transparency regarding the criteria for the identification of statements, steps for their decomposition, and the weighting of welfare effects. Therefore, the objective of this paper is to provide more clear and formalised guidelines and to take a major step forward by describing in more detail how to process scientific information in semantic modelling to enhance uptake by new modellers and to improve inter-modeller reliability and reproducibility. To this end, we specify the rules, supported by real-life examples, to explain the formalised decomposition and welfare weighting in semantic modelling.

## 2 General procedure of semantic modelling

**Figure 1:**
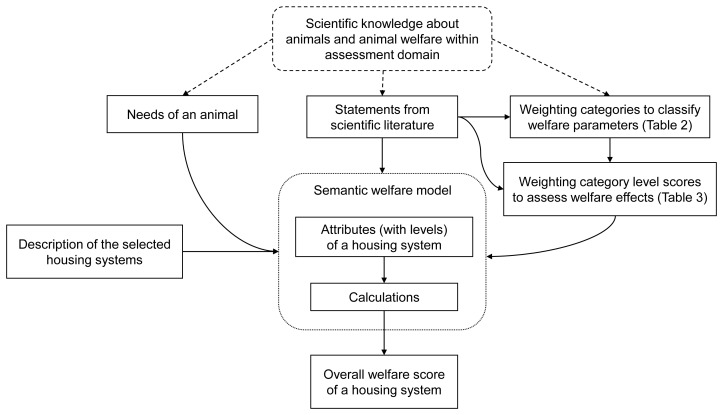
Schematic overview of concepts involved in semantic modelling for the purpose of animal welfare assessment (at the housing-system level).

The input of a semantic model consists of linguistically meaningful sections of the scientific literature (i.e. quotes), so called statements (Fig. 1). Statements typically describe the relationship between welfare input and output. The input generally consists of environment-based measures, also called attributes, dependent variables, or experimental treatments. The welfare output mainly refers to animal-based measures, also called independent variables or welfare measures. The if–then rule then typically reads as “If animals are exposed to environmental feature E compared to F then they show a (significant) increase or decrease in welfare measure M, implying that E is better or worse than F for welfare (other things being equal)”. Hence, attributes (e.g. enrichment material or stocking density), attribute levels (e.g. straw as enrichment material or a stocking density of 1 $m^{2}$ per pig) and their positive or negative effect on the animals' welfare (e.g. as measured by the number of skin lesions) can be extracted from scientific statements (see the glossary in Table 1). So-called weighting categories (WCats) classify different kinds of welfare measures into positive and negative measures. Three weighting categories indicate positive welfare, namely natural behaviour, preference, and (measures of) demand, whereas nine weighting categories denote reduced or negative welfare, i.e. (measures of) pain, illness, fitness, survival, aggression, stress physiology (HPA (hypothalamic–pituitary–adrenal) axis activation, SAM (sympathetic adreno-medullary) axis activation), abnormal behaviour, and frustration and avoidance (Table 2) (Bracke et al., 2002a).

To be able to calculate the weighting factor (WF) for each attribute, so-called weighting-category level scores (WCatLevScs) come into play (Tables 2 and 3). The WCats indicating positive welfare have positive WCatLevScs, while the negative WCats have negative scores. Moreover, the 12 above-mentioned WCats have been classified into relatively low-impact and high-impact categories based on their ability to reflect a lower or higher impact on the intensity, duration, and incidence as indicated by a welfare measure. The lower-impact WCats (i.e. presumed to be somewhat less important) use a WCatLevSc scale from ±1 to 3, the higher-impact categories (presumed to be more important) use a scale from ±1 to 5 (Table 3). Here, both the lower- and higher-impact WCats have three primary levels (i.e. respectively, ±1 , 2, 3 or ±1 , 3, 5) and a number of other possible scores besides these primary ones, especially at the low end of the scale from 0 to 1; see Part 1: Benthin et al., 2026). The higher-impact negative WCats are pain, illness, survival, frustration and avoidance, and HPA axis activation. They receive primarily negative WCatLevScs of -1 , -3 , or -5 . Demand is the only higher-impact positive WCat. It generates positive scores of +1 , +3 , or +5 . By contrast, lower-impact negative WCats that generate a negative primary WCatLevSc (i.e. -1 , -2 , or -3 ) include fitness, SAM axis activation, aggression, and abnormal behaviour. Natural behaviour and preference are lower-impact positive WCats that generate WCatLevScs of +1 , +2 , or +3 (Table 3; Bracke et al., 2002a). The WCatLevScs serve as input for the calculation of WFs for all of the attributes in the model. WFs are derived from the difference between the (sum of the) maximum positive and (the sum of the) minimum negative WCatLevScs for each WCat assigned to the best and worst levels of each attribute in the model, respectively (Table 1; see also Part 1: Benthin et al., 2026, Sect. 3).

Prior to calculating the WFs of attributes, at least two attribute levels, i.e. a best and worst level, need to be defined for each attribute in the model. Attributes need to apply across all housing systems within the assessment domain, and their respective attribute levels need to have a science-based ranking. For example, the attribute of floor type can have levels like fully slatted concrete floor, partly slatted concrete floor, and bedded floor. These levels are assigned only when scientific findings report differences, e.g. with respect to lameness, between these types of floor, such that one type is better than another. When the scientific evidence is inconclusive for a specific attribute, one or more attribute levels should be disqualified or combined to ensure that all attribute levels and their rankings have a scientific basis. For example, when the scientific evidence is inconclusive about whether “50 % slatted floor” is better or worse for animal welfare than “25 % slatted floor”, these attribute levels could be combined into the level “partly slatted floor” or “25 %–50 % slatted floor”. Subsequently, attribute-level scores (ALevScs) can be determined for each attribute by following the science-based ranking of their levels. For example, different levels of the attribute of floor type can be ranked from worst to best based on scientific studies reporting the short- and long-term effects of the different types of floor on animal welfare.

Finally, overall welfare scores are calculated as weighted-average ALevScs considering the fact that, together, all attributes in the model cover all welfare needs of the species of interest and all possible housing systems in the assessment domain (Bracke et al., 2002a; Fig. 1). A detailed explanation of the calculation steps for the overall welfare score is provided in Benthin et al. (2026).

**Table 2:** Overview of weighting categories (WCats, classes of positive and negative welfare measures) used in semantic modelling to assess animal welfare, modified after Bracke et al. (2002a). Column “Scale of WCatLevScs” (WCatLevSc: WCat level score) indicate which WCats are indicative of positive ( + ) or negative ( - ) welfare and whether the WCat has a relatively low or high impact (i.e. with a WCatLevSc of plus or minus 3 and 5 respectively; see Sect. 2 General procedure of sematic modelling).

Weighting category	Scale of WCatLevScs	Definition
Pain	0 to -5	Evidence of pain including lameness and skin lesions, e.g. from aggression or harmful social behaviour.
Illness	0 to -5	Evidence of health problems, including increased mortality due to illness but excluding lameness, skin lesions, and specific survival aspects.
Survival	0 to -5	Evidence of reduced survival related to physiological requirements (other than through specific health problems), e.g. longevity, deprivation of food or water, and a poor climate, but also predation and cannibalism, including mortality rates.
Fitness	0 to -3	Evidence of decreased fitness including (re)production effects (e.g. due to (heat) stress), physiological necessities, and associated risks (e.g. thermal control, metabolic compromises, hair or feather conditions, bone strength, feeding) but excluding specific survival aspects related to physiological requirements, HPA activation, and illness.
HPA	0 to -5	Evidence of activation of the HPA (hypothalamic–pituitary–adrenocortical) axis indicative of distress, e.g. elevated cortisol levels.
SAM	0 to -3	Evidence of SAM (sympathetic–adrenal–medullary) axis activation (indicative of negative affect), e.g. increased heart rate and (nor)adrenaline levels.
Aggression	0 to -3	Evidence of increased aggression (e.g. fights) including displacements or agonistic interactions, but excluding skin lesions (see pain).
Abnormal behaviour	0 to -3	Evidence of disturbed behaviour such as oral stereotypies, apathy, and disturbed sexual behaviour.
Frustration and avoidance	0 to -5	Evidence of behaviour indicative of fear or deprivation, including blocked behaviour or willingness to work to avoid a treatment.
Natural behaviour	0 to +3	Evidence of (a potentially positive reward from) behaviour as seen under (semi-)natural conditions, including time budgets and species specificity of that behaviour (e.g. behaviour that is very typical for a species (like grazing for cows, rooting and wallowing for pigs)), as well as behaviour that has positive welfare effects (e.g. play, dust bathing), but excluding normal behaviour of which the welfare effect in a specific context is debatable or unclear (e.g. lying or walking).
Preference	0 to +3	Evidence from preference test and behaviour under other-than-natural circumstances, including cognitive bias, rebound effects, and anticipation.
Demand	0 to +5	Evidence that animals are willing to exert effort to obtain a commodity, especially using operant conditioning (e.g. a weighted push-door to get access to a valued resource).

**Table 3:** Description of possible WCatLevScs (weighting-category level scores) and when to use them based on example words in a scientific statement.

WCatLevScs	When to apply a certain WCatLevSc	Examples of phrases used in statements to signal the magnitude of welfare effects
±1	The default WCatLevSc is ±1 . It is assigned when a statistically significant effect was found or mentioned/cited.	Statistical(ly) significant, effect, found, showed, is known, concluded, evidence, proof
±2 , 3, 5^a^	A higher WCatLevSc than ±1 (i.e. ±2 , 3, or 5) is given when strong phrasing or numerical data in the statement indicate a larger positive or negative welfare effect (e.g. based on wording or use of plurals).	High(ly), substantial, severe, very, strong(ly), most, much more, major, many, large, remarkable, profound, harsh, essential, important, chronic/cannibalism, outbreaks, typical species-specific behaviour patterns (e.g. rooting, wallowing, grazing)
±0.5	A WCatLevSc of ±0.5 is given when the statement contains weak phrasing or when no significant effects but tendencies (usually regarded to be p values > 0.05 and <0.1 ) were found.	May^b^, could, might, seems, risk, tendency/tended, perhaps
±0.01	The ±0.01 score is used to create an opposing score for what is potentially the worst or best level of an attribute mentioned in a statement, which can be overruled by future statements that show stronger results regarding that weighting category for a specific attribute level.	
0	A score of 0 is applied when there is no relation to an attribute level in the final database or when a statement reflects an expert opinion that has been replaced by later-found scientific literature for that attribute (level).	

^a^ Weighting categories are classes of welfare measures that indicate either positive ( + ) or negative ( - ) welfare (see the text and Table 2). Contrarily to the SOWEL model (Bracke et al., 2002a), the WCat of frustration and avoidance has been upgraded in ANyWEL to a relatively high-impact WCat that thus can generate a primary WCatLevSc of -1 , -3 , or -5 (it was upgraded because it includes negative demand; see Part 1: Benthin et al., 2026). ^b^ The word “may” is often used to express caution according to the scientific convention. We rely on the expertise of the modeller to decide whether “may” is used to indicate a tendency or weak statement (i.e. resulting in a score of ±0.5 ) or as scientific convention to express a statistically significant finding (resulting in a score of ±1 ).

Ultimately, the modelling principles and procedures developed for semantic modelling of animal welfare have been designed to generate the best-possible overall-welfare assessment based on all available information (at a certain point in time). This is achieved by systematically linking (as much as possible of) the available scientific literature about the animals and their living conditions (Bracke et al., 2002a). Interestingly, semantic models are not designed to be static constructs. They are intended to be updated periodically with the most recent scientific findings, and recent developments in AI are likely to facilitate this (Zhang et al., 2026).

## 3 Identifying scientific statements

**Figure 2:**
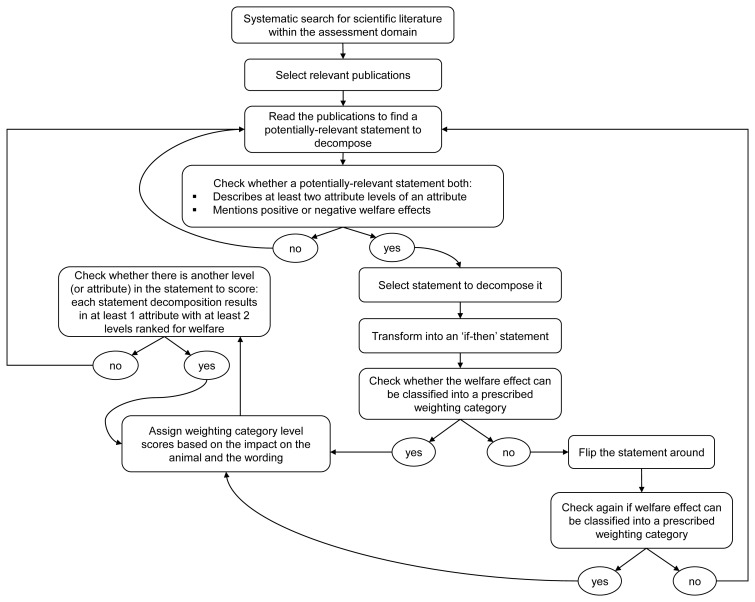
Schematic decision tree of steps taken to identify potential scientific statements and to score the attribute levels using the principles of semantic modelling. Note that reported welfare effects generate both welfare ranks of attribute levels (which generate attribute level scores) and weighting factors of attributes (via the link between the ranked attribute levels, the type of welfare measure, and so-called weighting categories and weighting-category level scores, as explained in more detail in the text).

In semantic modelling, a relational-database structure was developed with tables listing the scientific references, the scientific statements, and information about the location of the statement within the scientific reference in addition to the attribute(s); the attribute levels; the types of welfare measure; the associated WCats and WCatLevScs; and perhaps a note on, for instance, the interpretation of the statement (Bracke et al., 2001; Anonymous, 2001; see Supplement File S2 for an excerpt from a database file, and see Benthin et al., 2023). This enhances control over the data and allows for periodic updating when new information becomes available (Bracke et al., 2006). The first step in building such a database is to collect scientific statements for the species of interest within the defined assessment domain (i.e. range of housing systems) that meet the modeller's objective (Fig. 2). Literature sources include primarily peer-reviewed publications reporting empirical studies (including both original works and review papers) and scientific reports (e.g. expert-panel reports from the European Food Safety Authority). These sources can contain information on welfare and, in particular, describe relationships between environment-based treatments on the one hand and the behaviour, health status, and/or (stress-related) physiology of the species or animal type of interest on the other hand. Search criteria for literature should be clear, e.g. in accordance with PRISMA (Preferred Reporting Items for Systematic Reviews and Meta-Analysis; Page et al., 2021) as far as possible. Sources should preferably be available in the form of full text and should report statistically significant findings (Bracke et al., 2006). Papers published under an open-access licence make such sources more readily available (Muñoz-Tamayo et al., 2022). Also, other sources, such as textbooks, industry reports, and magazine articles, may be used if deemed appropriate, i.e. sufficiently trustworthy. In general, however, political or technical recommendations and legal standards should not be selected as they may not primarily rely on science. To fill knowledge gaps in the scientific literature, it is also possible to include statements reflecting expert opinions as another potential source of information (e.g. about new innovative housing concepts; see Part 1: Benthin et al., 2026).

The next step in semantic modelling is to identify from those literature sources the statements that describe the impact of experimental treatments on animal welfare. There is no minimum number of statements to extract; the modeller selects all statements that seem to be relevant and are feasible to process. The scientific statements are used to construct the welfare model by decomposing them into if–then relationships between the attributes of housing systems and reported welfare measures (Bracke et al., 2001). To extract the relevant information for semantic modelling, a semantic analysis, also called a decomposition of a statement, is needed (see also Sect. 4, “Decomposing scientific statements”). This is a language procedure that breaks down the meaning of phrases (i.e. statements) into segments based on logic. The criterion that flags a scientific statement as potentially worth considering for decomposition is the description of a positive or negative effect on the welfare of the relevant species of some property (attribute level) of a housing system within the assessment domain (Fig. 2).

For illustration purposes, here, we provide two examples of scientific texts that contain statements to be selected for making a welfare model. Example 1 is as follows:

In the farrowing pen, sows normally are housed individually in crates with a partly slatted floor with a heated resting area (solid floor with some substrate) for the piglets of at least 0.6 m^2^. In preference tests and operant conditioning tests, sows were highly motivated to farrow in pens with straw. (cited from Anonymous, 2001, p. 26)

The second sentence in example 1 meets the criterion of a scientific statement suitable for decomposition as it concerns farrowing pens with straw (as one attribute level) where the (positive) impact consists of the sow's high motivation for farrowing in pens with straw compared to pens without straw. This indicates that straw is important to fulfil (one of) the welfare needs of sows in farrowing pigs. Example 2 is as follows:

The effect of rearing with and without perches on the perch use of laying hens has been investigated by different authors. Hens reared without perches have difficulty using perches owing to low muscle strength, a lack of motor skills and the inability to keep balance, or they have impaired spatial skills, which are necessary for moving around in three-dimensional space. (cited from EFSA, 2015, p. 11)

Again, the second sentence in example 2 meets the criterion of a scientific statement that is useful in modelling layer welfare as it relates to the availability of perches (i.e. a design element of a housing system) during the rearing phase, whereas the (negative) impact on the laying hens' welfare arises from reduced fitness (i.e. indicating that the lack of perches during rearing negatively affects the birds' ability to use perches later in life due to low muscle strength).

## 4 Decomposing scientific statements

Once a scientific statement is deemed to be suitable for decomposition, the first step is to identify (i) the attribute, (ii) the levels of the attribute that are being compared, and (iii) the welfare effect that is being reported. The relationship between the attribute level and the welfare effect can be identified by transforming the original statement into an if–then rule (Bracke et al., 2001, 2002a; Fig. 2). In this procedure, the meaning of both the original statement and the if–then statement remains logically equivalent. The if–then statement can be formulated as follows:

If animals (i.e. the species of interest) are subjected to attribute level L1 of attribute A as compared to its level L2 then there is a (significant) effect (i.e. increase or decrease) on welfare measure M that belongs to weighting category WCat and has a magnitude of WCatLevSc S. (see Sect. 4.2 in Part 1: Benthin et al., 2026)

By specifying the if–then relationship between the environmental characteristics and the WCats, the essential elements for semantic modelling are revealed. This can be demonstrated using the two previous example statements: considering example 1, the statement “In preference tests and operant conditioning tests, sows were highly motivated to farrow in pens with straw” would be converted into “If sows are kept in farrowing pens with straw compared to pens without straw (where A denotes presence of nest-building material in the farrowing pen, L1 denotes straw available, and L2 denotes no straw available) then sows will show a preference (WCat of preference) and are willing to work for (WCat of demand) the pens with straw”. Considering example 2, the statement “Hens reared without perches have difficulty using perches owing to low muscle strength, a lack of motor skills and the inability to keep balance, or they have impaired spatial skills, which are necessary for moving around in three-dimensional space” would be transformed into “If hens are reared without perches (where A denotes perch availability, and L1 denotes not available) then they have lower muscle strength (WCat of fitness), lack of motor skills (WCat of fitness), an inability to keep balance (WCat of fitness), or impaired spatial skills (WCat of fitness) compared to hens reared with perches (L2 denotes perches are available)”.

Sometimes the welfare effect in the statement appears to be mismatched with the positive or negative WCat as defined previously (Bracke et al., 2002a). For example, a statement may present a positive welfare effect (e.g. improved claw health in treatment L1 compared to in L2), whereas the available WCat (pain) describes only negative effects (e.g. increased lameness). The solution here is to flip the statement around as it were (i.e. negate it) when the if–then rule is deduced from the original statement. Thus, the seemingly positive effect mentioned in the original statement (i.e. improved claw health in L1, which, in fact, was only a reduction in negative welfare) is converted into a negative effect in the if–then statement (i.e. increased lameness in treatment L2 compared to in L1). This negative effect can then be classified (and scored) using the negative WCat of pain. The following example illustrates this procedure again with treatment L1 being “grazing on pasture” and L2 being “no grazing possibility available”:

Grazing is known to benefit claw health (less severe disorders and better recovery) and to reduce stereotypies and aggression in the herd. (cited from Cornelissen at al., 2009, p. 7)

The solution here for using the negative WCats to weight “improved claw health”, “reduced stereotypies” and “reduced aggression” is to flip the treatments around as it were when the original statement is transformed using the if–then rule. This generates the following formulation: if … no grazing opportunities are available (where A denotes pasture access, and L1 denotes no pasture access) then … more claw problems (WCat of pain), more stereotypies (WCat of abnormal behaviour), and more aggression (WCat of aggression) are known to occur compared to when cattle have grazing opportunities (L2 denotes with pasture access with grazing opportunities) available.

## 5 Assigning weighting-category level scores (WCatLevScs)

Decomposing a scientific statement through the conversion into an if–then statement helps to identify the attribute levels, as well as their welfare rank (and their attribute level scores, ALevScs). Subsequently, a WCatLevSc can be assigned by classifying the reported welfare measure and assigning a score for the degree to which welfare was affected according to (the meaning of) the scientific statement. The WCatLevScs are assigned by the modeller based on the incidence, duration, and intensity of the reported welfare effect, i.e. how many animals are affected, for how long, and how severely (Willeberg, 1991; Fig. 2, Tables 2 and 3). As mentioned earlier, there are positive and negative WCats that have either a relatively low or high impact (with primary WCatLevScs of ±1 , 2, 3 and ±1 , 3, 5, respectively; Bracke et al., 2002a). Modellers should take note of the use of specific wordings in a statement (e.g. chronic or severe) that say something about the incidence, duration, and/or intensity of the welfare problem when assigning a WCatLevSc. By default, a score of ±1 is given for statistically significant (or equivalent) findings. A higher score of ±2 , 3, 5 is used for a strong(er) welfare effect, as indicated by the welfare measure (i.e. classified as a relatively low- or high-impact WCat) or based on the use of specific terms or phrases in the scientific statement that is being analysed. We listed examples of such phrases that are typically associated with a WCatLevSc of ±1 and those associated with the higher scores ±2 , 3, 5 (Table 3). The example phrases mentioned in the third column of Table 3 (i.e. phrases like “chronic”, “severe”, and “highly”) are used to generate higher WCatLevScs based on the general rule of thumb that the authors of the statements have used these words only when justified. Consequently, these “power terms” are taken to indicate a strong(er) positive or negative impact of a certain attribute level on the animal's welfare. Besides specific phrases, modellers can also make use of numerical data about the incidence, duration, and/or intensity of a welfare problem. The following example illustrates this:

[adventitious] Bursitis was found to affect 95.5 per cent of pigs reared without straw compared with 3.75 per cent of pigs reared with straw (Smith & Smith 1990). (cited from Arey, 1993, p. 240)

Here, almost all pigs were affected by a condition causing lameness when housed on concrete floors compared to straw bedding; hence, the attribute level of concrete floor for the attribute of floor type would receive a maximum WCatLevSc of -5 for the relatively high-impact WCat of pain.

Besides the two standard scales (plus or minus 1, 2, 3 and 1, 3, 5) for the two kinds of WCats (i.e. with a relatively low and high impact, respectively), we suggest using a lower proxy score of ±0.5 . That score should be used for uncertain statements based on weak phrasing (Table 3) or tendencies to reduce the impact of publication bias (Benthin et al., 2026). This may be useful since there is discussion within the scientific community about null-hypothesis significance testing based on arbitrary P -value thresholds and the use of the term “statistical significance” (Muff et al., 2022). Furthermore, a score of 0 was used for statements that needed to be discarded because they had been updated by new statements (such as an expert opinion on an innovative new housing system that is replaced by new scientific findings). Statements may also be discarded from the calculation of WFs when they end up not having a relation to any of the final attribute levels in the model. Moreover, when WCatLevScs are assigned, it is important that all attribute levels within the statement receive a scoring. Thus, the potentially best and worst level of the attribute must receive, respectively, a minimum of one positive and one negative WCatLevSc (Fig. 2). This is a practical requirement to enable the calculation of the overall welfare score (OWSc) using the semantic model (Bracke et al., 2002a; Part 1: Benthin et al., 2026).

An attribute level can receive several WCatLevScs based on one or more statements, which affect the WF for that attribute. In case two or more statements report opposite welfare effects, the WCatLevScs could cancel each other out. In addition, the welfare effect of the treatments (attribute levels) compared within the scientific statement may not always be clear by means of the specified WCats. For instance, we present the following statement: “In preference tests and operant conditioning tests, sows were highly motivated to farrow in pens with straw” from example 1. Here, the word “highly” specifies a strong welfare effect. In addition, welfare measures from both preference and motivation tests (stated also in plural) are mentioned. Hence, a higher WCatLevSc of +2 for the WCat of preference (with the scale +1 , 2, 3) and +3 for the WCat of demand (with the scale +1 , 3, 5) can be assigned to the attribute level “straw available” of the attribute “type of nest-building material”. Since feed is considered to be the gold standard in motivation tests (Dawkins, 1988) and because the scientific statement does not compare access to straw with access to feed, no maximum WCatLevScs (i.e. +3 and +5 , respectively) are assigned. In this statement, the attribute level “straw available” is compared to the level “no straw available”. However, the statement (example 1) does not report a negative welfare effect of not having straw available in the farrowing pen. This attribute level (no straw available) could potentially be the worst level of the attribute “type of nesting material”. In the process of decomposing statements, it may not be known whether more statements will be found about these attribute levels. Therefore, we suggest assigning a WCatLevSc of ±0.01 (i.e. near to 0) to ensure that there is always a difference between the best and worst level of each attribute (see also our companion paper by Benthin et al., 2026, and Table 3). By assigning a WCatLevSc close to 0 (i.e. 0.01) to the complementary attribute level rather than not having assigned any value, the semantic model as implemented in a relational database can still calculate the WFs of all attributes. Consequently, the attribute level “no straw available” in this statement receives a score of -0.01 for the WCat of frustration and avoidance (i.e. assuming that nest-building behaviour is blocked in sows when no nest-building material is available). In the process of further statement decomposition, the modeller is very likely to encounter one or more other statements that more clearly show that the attribute level “no straw available” can cause frustration in farrowing sows. Then, the -0.01 score is overruled by higher (more negative) WCatLevScs. An example is the next statement:

At farrowing, sows are normally confined in crates without bedding. These conditions can lead to acute signs of stress as shown by both the sow's behaviour (Baxter, 1982; Schouten, 1987) and physiology (Baxter and Petherick, 1980; Metz and Oosterlee, 1981; Kilgour and Dalton, 1984; Vestergaard, 1984). Much of the frustration may be caused by the lack of materials to build a nest. (cited from Arey, 1993, p. 238)

The decomposition of this statement will replace the complementary score of -0.01 with a WCatLevSc of -1 (WCat of frustration and avoidance) for the attribute level “no straw available” in the farrowing pen.

Once all of the selected sources have been read and all of the relevant statements have been identified and decomposed, the names and descriptions of the attributes and attribute levels are to be revisited to finalise the database. The modeller needs to ensure that multiple statements referring to the same attribute and/or attribute level are labelled with identical names. This will consolidate the science-based ranking of attribute levels, including the best and worst level of each attribute, such that attribute level scores and WFs can be assigned properly. For the ranking of attribute levels, the modeller has to review the WCatLevScs of each attribute level and (i) sum up the lowest WCatLevScs for each negative WCat and (ii) sum up the highest WCatLevScs for each positive WCat. In this way, the modeller can rank the attribute levels of each attribute from the best to worst level. Here, the best level has the highest sum of the most positive WCatLevScs (and, if any, also added to that the most negative WCatLevScs) per WCat. The worst level has the lowest sum of the most negative WCatLevScs per WCat (and, if any, also added to that the most positive WCatLevScs; see also Part 1: Benthin et al., 2026). Afterwards, the model can be used to calculate the overall welfare scores for housing systems within the assessment domain, e.g. to generate welfare scores of benchmark systems (see Benthin et al., 2026, and Bracke et al., 2002a, for this procedure).

## 6 Final remarks

Overall, statements collected from the scientific literature and, if needed, from interviews with experts are key for semantic modelling of animal welfare. To enhance the scientific reproducibility and transparency of overall welfare assessment using a semantic welfare model, we formalised (i) the relevant scientific knowledge sources, (ii) the criterion that flags a potentially meaningful scientific statement for semantic modelling, and (iii) described the if–then procedural rules for the decomposition of statements. This may further explain why we believe semantic modelling is bio-logical: it is based on an understanding of (the semantics of) the biology of the animals, as well as on logic. As to logic, semantic modelling implies something like this:

If n scientific statements (S1, … Sn) are known then, since S1 is true, S2 is true, … and Sn is true, for each S there is an attribute level L1 that is better for welfare than L2 as measured by welfare measure M, and if all of that is the case then we can derive i attributes (A1, … , Ai), each with ranked levels L_worst_ … L_best_ and weighting factors (WF1, …, WFi), such that overall welfare scores (OWScs) can be calculated for all housing systems in the assessment domain based on the available science (i.e. the collected statements) by calculating weighted average attribute level scores (which are based on the attribute level ranks and on the intensity, duration, and incidence of the reported welfare effects).

To further stimulate (this kind of) transparency, we strongly recommend that, for each welfare model, the scientific statements with their respective decompositions should be made publicly available (e.g. Benthin et al., 2023). As part of the decomposition of scientific statements, there are two classification tasks that also involve normative judgements: on the one hand, the identification of attributes and attribute levels in statements and, on the other hand, the assignment of WCatLevScs to reported welfare effects as measured in scientific studies. Yet, the semantic-modelling methodology formally separates the descriptive and prescriptive elements in the meaning of scientific welfare statements. Moreover, it logically restricts itself to the descriptive task (aiming for a best-possible, transparent, and factual welfare assessment based on science). Hence, it uses norms common in science and the principles of formal logic, e.g. by extracting if–then rules from scientific statements. Additionally, it uses the bio-logical meaning of the underlying scientific information based on the common conceptual framework for science-based animal welfare assessment (Anonymous, 2001). The modeller aims to ensure that the statements and their decomposition are logically and semantically equivalent. This approach requires rather strict logical thinking and intends to avoid bias and circular reasoning (Bracke et al., 2006). Nevertheless, this part of semantic modelling may be disputed given the partly subjective interpretation of the meaning of the underlying scientific information (Stien et al., 2013; Bracke et al., 2006). This subjectivity is decreased when the quality and the number of scientific statements and publications are increased. More solid data reduce the freedom of the interpretation of the data and enhance the state-of-the-art consensus in science. Moreover, we introduced a WCatLevSc score of ±0.5 for results exhibiting only statistical tendencies or originating from studies of marginal quality (see also Benthin et al., 2026). Incorporating these lower-weighted statements enhances model robustness and helps to mitigate publication bias by capturing findings that would otherwise be excluded. Users of semantic models should be aware that attributes may vary in terms of the number and quality of the scientific statements they are based on. This can be mitigated by the recommended transparency, as well as by the fact that limited scientific evidence will generally result in a lower WF (i.e. in attributes of relatively minor importance). Without in any way denying that further improvement can and should be made, the semantic-modelling procedures and models are sufficiently scientifically sound: firstly, in the sense of being published in peer-reviewed articles; secondly, in being, in principle, empirically testable (e.g. with the body of scientific knowledge); and, thirdly, in being designed to take the modeller's point of view out of the equation as much as possible (Bracke, 2008). The guidelines presented in this paper are likely to further reduce the level of subjectivity and risk of mistakes by having a more formalised and standardised process of semantic modelling as we are the first to spell out the crucial steps of semantic modelling in such detail. This will aid future modellers in gaining a better understanding of the approach and will improve the reproducibility of future welfare models. Recently, Zhang et al. (2026) built an AI system that can automatically read scientific papers and pull out statements related to animal welfare. This study compared the datasets with scientific statements compiled using the ANyWEL model framework (Benthin et al., 2023, 2026, Part 1) to those compiled for SOWEL, COWEL, SWIM, and FOWEL. The authors concluded that the AI system performed better on the ANyWEL subset than on the other four subsets of scientific statements. The standardisation in ANyWEL through the guidelines presented in this paper likely reduced ambiguity considerably, making relevant statements easier to identify automatically (Zhang et al., 2026). Future cohort studies are needed to confirm an increase in inter- and intra-observer reliability due to the new guidelines. Moreover, we recommend the training and monitoring of intra- (and inter-)modeller agreement through, for instance, regular review sessions in which the modeller revisits scientific statements and repeats the decomposition and scoring activities involved in the modelling process. By these means, modellers can check their own and each other's consistency in deriving relevant information from scientific statements. Particularly, training and reflection on the use of certain power terms (e.g. “severe”, “much more”, “chronic”) that can be decisive for a higher WCatLevSc could help modellers to improve their consistency and reliability. Future efforts should be made to further formalise the derivation of attributes and their levels based on the scientific statements. In particular, the level of detail of selected attributes in the model is an aspect that requires further clarification.

## 7 Conclusions

This work provides a set of essential formalised rules regarding how scientific literature can be used to build a semantic welfare model by spelling out the process of semantic modelling. We specified how scientific information can be retrieved, recorded, decomposed, and weighted using semantic-modelling principles. The explanations and recommendations presented in this paper's guidelines could be an important step forward in making the use of scientific literature for integrated welfare assessment more transparent and standardised at the inter-modeller scale.

## Supplement

10.5194/aab-69-383-2026-supplement The supplement related to this article is available online at https://doi.org/10.5194/aab-69-383-2026-supplement.

## Data Availability

Data can be obtained upon request.
