# Supplementary material for: Semantic modelling of animal welfare explained – Part 2: The basis of welfare weighting and usage of scientific information
Source: Arch Anim Breed. 2026 Jul 16;69(3):383–96. doi: 10.5194/aab-69-383-2026 (PMC13480438; doi:10.5194/aab-69-383-2026)
Supplement: Supplementary file 1 [file aab-69-383-2026-supplement.zip › aab-69-383-2026-supplement-title-page.pdf]

Supplement of Arch. Anim. Breed., 69, 383–396, 2026
https://doi.org/10.5194/aab-69-383-2026-supplement
© Author(s) 2026. CC BY 4.0 License.

Open Access
Archives Animal Breeding

Supplement of

Semantic modelling of animal welfare explained – Part 2: The basis of welfare weighting and usage of scientific information

Margret L. Vonholdt-Wenker et al.

Correspondence to: E. Tobias Krause (tobias.krause@fli.de)

- aab-69-383-2026-supplement-title-page.pdf
- Vonholdt-Wenker et al Supplementary Material S1_R2ed.docx
- Vonholdt-Wenker et al Supplementary Material S2_R2ed.xlsx

The copyright of individual parts of the supplement might differ from the article licence.
