# Supplementary material for: Semantic modelling of animal welfare explained – Part 2: The basis of welfare weighting and usage of scientific information
Source: Arch Anim Breed. 2026 Jul 16;69(3):383–96. doi: 10.5194/aab-69-383-2026 (PMC13480438; doi:10.5194/aab-69-383-2026)
Supplement: Supplementary file 1 [file aab-69-383-2026-supplement.zip › Vonholdt-Wenker et al Supplementary Material S1_R2ed.docx]

**S1.1 Introduction**

During the development of the ANyWEL model framework (Benthin et al., Submitted) and the presented formalised rules for the decomposition of scientific statements, we conducted an inter- and intra-modeler agreement assessment to test and further improve the recommendations. Accordingly, three modelers (named Modeler 1, 2, and 3) read scientific literature and identified scientific statements for semantic modelling. The modelers were all familiar with semantic modelling and statement extraction. Each modeler was assigned to a different focal taxon for their original assessments (i.e. Modeler 1: poultry, Modeler 2: pigs, Modeler 3: cattle). Weeks after the original assessments were conducted, reassessments were made by randomly selecting a number of statements from poultry (i.e. the focal taxon of Modeler 1) to measure intra- and inter-modeler reliability. For each statement (see Table S1 below), the decomposition was re-done to extract again the attributes, attribute levels, weighting categories (WCat), type of welfare measure (Type), weighting category level scores (WCatLevScs), and needs (see Table 1 in manuscript for a glossary). The aim of the intra- and inter-modeler reliability assessment was to see how reliable the statement decomposition was within and among different modelers.

**S1.2 Method**

**S1.2.1 Reliability assessment**

To demonstrate an example of inter- and intra-modeler reliability analysis in semantic modelling, we used 10 statements about poultry welfare that were included in the ANyWEL dataset (see Table S1; Benthin et al., 2023 and Benthin et al., Submitted). The additional scoring scale for WCatLevScs as suggested in the ANyWEL framework described in this manuscript and our companion paper (Benthin et al., Submitted) was included for the inter- and intra-modeler reliability analysis. The decomposition of those statements led to a certain number of rows in a table based on the extracted information. Each row contained information about i) attributes, ii) attribute levels, iii) WCats, iv) Types, v) WCatLevScs, and vi) needs. While WCatLevScs were analysed separately as numerical variable, the five remaining nominal variables were analysed by categorizing their (intra- and inter-modeler) agreement as follows for each cell in the table (i.e. the number of cells = number of rows (identified attributes) multiplied by the non-numerical category entries per row (i.e. 5 as mentioned above)):

- No changes (marked in green): No changes in wording of the respective cell had taken place. If more than one keyword or term was written in the respective cell, none of them differed.
- Changes in wording without changes of meaning (marked in yellow): Wording of at least one of the cells’ keyword or terms, was altered in typing or orthography, but the meaning remained the same, e.g. “Perch height” in comparison of “Height of perches”.
- Changes in meaning (marked red): The meaning of the keywords or terms in the respective cell had been changed, or deletion/addition had taken place.
- Changes in cells with several keywords or terms, where some were changed in their meaning (i.e. red) and some remained unchanged in their meaning/or even in their exact spelling (marked in pink).

For both the intra- and inter-modeler reliability analysis, the three modelers provided written assessments. These were, as above described, categorised by a fourth person also familiar with semantic modelling procedures. Thus, decisions whether changes e.g. in meaning appeared or not, were not made by the modelers themselves.

***Table S1.*** Poultry statements used for intra- and inter-modeler assessment. Stat-ID: statement identification number.

| **Species** | **Stat-ID ^a^** | **Statement** |
| --- | --- | --- |
| Poultry | 89 | The incidence of foot pad dermatitis paralleled high litter moisture and caking scores, with chipped pine wood, chopped straw, cotton-gin trash, and pine shavings showing the highest severity scores and mortar sand and ground door filler showing the lowest (Bilgili et al., 2009). |
| Poultry | 166 | In addition, the risk of vent pecking was increased in single-tier systems when perches were placed at more than 50 cm above the slats, compared with less than 50 cm or no perches (Lambton et al., 2015). |
| Poultry | 169 | Brendler et al. (2014) found that nearly all birds roosted on perches at heights of 90cm or above. |
| Poultry | 214 | The contact area of foot pads was lowest on round perches, followed by oval and then square perches. |
| Poultry | 227 | In conclusion, the experts assumed that hens like to have the opportunity to get as high as possible above ground level, and stated that this level should be accessible by a series of small sized steps (e.g. 40 cm). |
| Poultry | 388 | On the other hand, Leone and Estevez (2008) used a different method, the nearest neighbour distance, of much smaller groups of 10-20 broilers in small pens to argue that birds were only attracted to each other at very low stocking densities (2.2-3.4 birds/m^2^) and were repelled at stocking densities higher than this. |
| Poultry | 395 | For instance, red light can increase activity level [17] and fear in broilers [18], while in layers it is linked to an increase in productivity [16,19]. A combination of red and white light appears to reduce stress levels in layers [20]. |
| Poultry | 404 | Other general positive effects related to provision of UV-A light, such as reduced fearfulness in broilers [32] and in layers [12,13,33] have been found. |
| Poultry | 582 | Furthermore, poorly drained areas of the range can result in pools of water which hens may drink from, increasing their exposure to bacteria such as *E. coli* and *Brachyspira* (Lister and van Nijhuis, 2012). Such pathogens can lead to significant mortality in a flock. |
| Poultry | 763 | As floor space decreases, within a range of 650–300 cm^2^/hen, biological function generally decreases, leading to either higher mortality, lower egg production and body weight or poorer feed conversion (Hughes, 1983; Sohail et al., 2004). |

^a^ References: 89: De Jong et al. (2012) EFSA Journal, p.32; 166, 169, 214, 227: EFSA (2015) EFSA Journal, p. 24, 36, 12, 42 (respectively); 388: Dawkins (2017) Advances in poultry welfare, p. 233; 395 and 404: Wichman et al. (2021) Animals, p. 2 and 12 respectively; 582: Sandilands (2020). Understanding the behaviour and improving the welfare of chickens, p. 387; 763: EFSA (2023) EFSA Journal, p. 70.

**S1.2.2 Statistical analysis**

Inter- and intra-modeler reliability for nominal variables was calculated as percentage of agreements. Therefore, the number (#) of cells that had “no changes (green)” and the number of cells with “changes in wording without changes of meaning (yellow)” were summed up as correctly captured by the modeler. The correctly captured cell counts were brought into relation to the total number of cells for the statements, thus number of rows multiplied by number of columns (i.e. 5) following Eq. (S1):

$Rb=\left( \frac{\sum_{modeller i} cells "no changes"\pm\sum_{modeller i} cells "changes in wording but not meaning"}{\sum_{modeller i} rows*5} \right)*100$	(S1)

where Rb = the percentage of agreement per modeler. The percentage of disagreement per modeler (DRb) was calculated following Eq. (S2):

*DRb*=100-*Rb*	(S2)

Inter- and intra-modeler reliability for the WCatLevScs was assessed using Spearman rank correlations (rS) as they were non-normal distributed numerical values. For this purpose, SigmaPlot 14.0 (Systat Software Inc.) was used.

**S1.3 Results**

**S1.3.1 Intra-modeler reliability**

A total of 45 rows and 5 columns (i.e. attributes, attribute levels, WCats, Type of welfare measure, and needs) led to 225 cells that were analysed for the intra-modeler agreement for poultry (i.e. modeler 1, the assigned poultry data extractor). Green and yellow categories were merged, as in both the meaning of the decomposition remained. For the intra-modeler reliability, 80% of the cells were reliably captured in the intra- modeler assessment (Table S2). The correlation between the WCatLevScs in the intra-modeler assessment was r_S_ = 0.97, p<0.0001 (Spearman rank correlation, N = 42).

***Table S2.*** Outcomes of the intra-modeler reliability assessment for Modeler 1.

| No changes | Changes without changes of meaning | Changes in meaning | Changes in cells with several terms, where some were changed in their meaning (i.e. red) and some remained unchanged in their meaning / or even in their exact spelling |
| --- | --- | --- | --- |
| 133 cells | 48 cells | 30 cells | 14 cells |
| 59.11 % | 21.33 % | 13.33 % | 6.22 % |
| In total 181 cells, thus Reliability is Rb = 80.44% | | In total 44 cell, thus DRb = 19.55%) | |

**S1.3.2 Inter-modeler reliability**

That original statement decompositions are substantially recaptured by the same modeler has been shown above (see Table S2), with an agreement in more than 80% of the cells. Whether these original statement decompositions were similarly executed by the other modelers was the subject of the inter-modeler reliability. Here, we tested how well the original statement decompositions of Modeler 1 matched to the decomposition of the two other modelers (i.e. Modelers 2 and 3). So, the decomposition of Modelers 2 and 3 regarding the 10 poultry statements (Table S1) were compared to the original decomposition of Modeler 1.

The assessment of the inter-modeler agreement between Modeler 2 and the original statement decomposition (made by Modeler 1) led to a total of 48 rows with 5 columns each (i.e. attributes, attribute levels, WCats, Type of welfare measure, and needs), which resulted in 240 cells that were analysed for the inter-modeler agreement for poultry. The inter-modeler agreement of Modeler 2 with the original assessment of Modeler 1 was about 40% (Table S3). The correlation of the WCatLevScs for the inter-modeler assessment between Modeler 2 and the original statement decomposition of Modeler 1 was r_S_ = 0.59, p<0.0003 (Spearman rank correlation, N = 34).

***Table S3.*** Outcomes of the inter-modeler reliability assessment for Modeler 2 *vs*. original statement decomposition of Modeler 1.

| No Change | Changes without changes of meaning | Changes in meaning | Changes in cells with several terms, where some were changed in their meaning (i.e. red) and some remained unchanged in their meaning / or even in their exact spelling |
| --- | --- | --- | --- |
| 64 cells | 33 cells | 127 cells | 16 cells |
| 26.66 % | 13.75 % | 52.92 % | 6.66 % |
| In total 97 cells, thus Reliability is Rb = 40.42% | | In total 143 cells, thus DRb = 59.58% | |

The assessment of the inter-modeler agreement between Modeler 3 and the original statement decomposition (of Modeler 1) led to a total of 52 rows with 5 cells each (i.e. attributes, attribute levels, WCats, Type of welfare measure, and needs), which resulted in 260 cells that were analysed here for the inter-modeler agreement for poultry. The inter-modeler agreement between Modeler 3 and the original statement decomposition was 54% (Table S4). The correlation between the WCatLevScs for the inter-modeler assessment between Modeler 3 and the original assessment of Modeler 1 was r_S_ = 0.969, p<0.0001 (Spearman rank correlation, N = 35).

***Table S4***. Outcomes of the inter-modeler reliability assessment for modeler 3 *vs.* the original statement decomposition.

| No Change | Changes without changes of meaning | Changes in meaning | Changes in cells with several terms, where some were changed in their meaning (i.e. red) and some remained unchanged in their meaning / or even in their exact spelling |
| --- | --- | --- | --- |
| 82 cells | 59 cells | 110 cells | 9 cells |
| 31.53 % | 22.69 % | 42.31 % | 3.46 % |
| In total 141 cells, thus Reliability is Rb = 54.23% | | In total 119 cells, thus DRb = 45.77% | |

**S1.4 Conclusion**

So, all in all, both inter-modeler reliability analyses showed a moderate agreement of cells between 40% and 54%, and a weighting category level score (WCatLevSc) correlation of at least r_S_>0.59 compared to the original statement decomposition of Modeler 1. Nonetheless, intra-modeler agreement was substantially high with more than 80% agreement of cells and a WCatLevSc correlation r_S_=0.97.
